# Conventional and Unconventional Antimicrobials from Fish, Marine Invertebrates and Micro-algae

**DOI:** 10.3390/md8041213

**Published:** 2010-04-14

**Authors:** Valerie J. Smith, Andrew P. Desbois, Elisabeth A. Dyrynda

**Affiliations:** 1 Scottish Oceans Institute, formerly the Gatty Marine Laboratory, University of St Andrews, St Andrews, KY16 8LB, Scotland, UK; 2 Biomedical Sciences Research Complex, University of St Andrews, St Andrews, KY16 9ST, Scotland, UK; 3 Centre for Marine Biodiversity and Biotechnology, School of Life Sciences, Heriot Watt University, Edinburgh, EH14 4AS, Scotland, UK

**Keywords:** amphipathicity, antimicrobial peptides, fatty acids, innate defence, pigments

## Abstract

All eukaryotic organisms, single-celled or multi-cellular, produce a diverse array of natural anti-infective agents that, in addition to conventional antimicrobial peptides, also include proteins and other molecules often not regarded as part of the innate defences. Examples range from histones, fatty acids, and other structural components of cells to pigments and regulatory proteins. These probably represent very ancient defence factors that have been re-used in new ways during evolution. This review discusses the nature, biological role in host protection and potential biotechnological uses of some of these compounds, focusing on those from fish, marine invertebrates and marine micro-algae.

## 1. Introduction

Eukaryotic organisms, especially those from the marine environment, represent a rich hunting ground for the discovery of novel natural microbicidal agents. Not only do the seas contain representative species of nearly all the main animal taxa and a huge diversity of photosynthetic organisms, but they are also microbe-rich. Accordingly, over evolutionary time, marine eukaryotes have developed a plethora of anti-infective molecules and strategies by which they protect themselves against prokaryotic and viral attack. It is no surprise, therefore, that much effort has been expended in identifying and characterizing antimicrobial factors from these organisms. Of the many reports that have been published so far, the majority have been concerned with small peptides that are often cationic, amphipathic and encoded by single genes. Sequence information obtained from various animals for over 1,500 of such proteins, known popularly as antimicrobial peptides (AMPs), are now lodged on databases or published in the literature. There are also some review articles [1–3]. However, in addition to these conventional or ‘professional’ antimicrobials are a variety of other factors produced by eukaryotic organisms that are not usually regarded as immune-relevant molecules but, nonetheless, have potent antimicrobial activities. Such factors may be molecules that serve other biological roles, such as maintenance of structural integrity or signalling. Alternatively they may be fragments derived from these proteins or lipids. Because they are unlikely to encounter opportunistic or invasive micro-organisms, these factors are seldom included in review articles on antimicrobial peptides or innate immunity. In an important early review of animal antimicrobial peptides, Hans Boman noted the existence of such ‘unconventional’ antimicrobial factors but did not include them within his four main categories of proteins distinguished on the number and type of certain residues and/or main structural features [4]. Rather, he combined these factors together as a miscellaneous group that he designated as ‘peptides or fragments derived from proteins of other function [4]. Since Boman’s paper was published the number of molecules now known to disrupt or kill bacterial cells has grown considerably.

These unconventional antimicrobials have structural motifs that are biologically useful and which have been conserved during evolution. There are occasions when they might be brought into intimate contact with potentially harmful micro-organisms, and, thus augment the more conventional reticulo-endothelial or mucosal immune networks. Circumstances where this might happen include mechanical trauma, tissue damage or controlled cell death. These are especially important for single-celled eukaryotes, simple-bodied marine invertebrates and deuterostomes that lack adaptive immunity mediated through lymphocytes and immune memory. Accordingly, the present article is aimed at describing some of these unconventional anti-infective agents using examples from fish, invertebrates and micro-algae, especially those from the sea of economic or biotechnological importance. However, mention is also made of antimicrobial agents from some freshwater organisms, especially fish and shellfish, where certain features of their microbicidal factors are relevant to similar factors in marine species or happen to have been studied more extensively in aquatic types.

## 2. Conventional AMPs

Conventional AMPs are broadly defined as small (<10 kDa, or ~12–50 amino acids) cationic, amphipathic peptides [5]. AMPs are regarded as dedicated, ‘professional’ antimicrobial molecules, commonly located where they are most likely to be needed, *i.e.*, in peripheral blood cells or epithelial (mucosal) surfaces. In general, AMPs are synthesized as precursors containing the active peptide segment, which is released by proteolytic processing, usually upon the removal of an anionic propiece [4]. AMPs have been described from diverse taxa throughout the animal kingdom although, amongst marine and aquatic species, their most frequent identification has been in fish and shellfish (Table 1), reflecting the high level of interest given to economically important animals.

The first AMPs from aquatic animals were identified as proteins on the basis of their antimicrobial activity during biochemical purification [12,25,28], while more recently, putative novel AMPs have been found by molecular techniques such as expressed sequence tag (EST) analyses and gene cloning [32,33]. A definitive classification scheme still needs to be agreed but most authorities recognise at least 3 main classes of conventional AMPs based on their secondary structure, namely: linear α-helical peptides; cysteine-rich peptides containing β-sheets and disulphide bonds; certain amino acid enriched peptides (Table 2). As might be expected, given their extremely diverse sources, not all peptides necessarily fit into clear categories and therefore, for the purposes of this review, these are grouped separately into a further miscellaneous category (Table 2).

For all conventional AMPs, features such as size, charge, conformation/secondary structure, hydrophobicity and amphipathicity are critical in determining antibacterial activity and modes of killing and have been well reviewed previously [34]. However, the precise mechanisms of peptide-membrane interactions and bacterial killing for many AMPs are often unconfirmed, particularly for those from marine sources. In general, killing relies on the initial attraction of the peptide to the bacterial surface and its subsequent attachment and interaction with the cytoplasmic membrane [35,36]. Attraction is largely mediated by the electrostatic interaction between the positively-charged peptides and the negatively-charged molecules of the bacterial cell wall. Following this, killing is dependent on AMP insertion into, and permeabilization of, the cytoplasmic membrane *via* various mechanisms [35,36]. Three main mechanisms have been proposed to explain this, which are the barrel-stave, toroidal pore and carpet models. These have been described and illustrated elsewhere [34]. The barrel-stave model describes pore formation by the alignment of the hydrophobic peptide regions with the bacterial membrane lipid head groups, with the AMP aligned parallel with the membrane surface [37]. Repositioning of the AMP perpendicular to the membrane creates the pore, of which the hydrophilic regions of the AMP form the pore interior [37]. However, this model has been confirmed for only a small number of peptides (e.g., alamethicin from the fungus, *Trichoderma viride* [37]). The formation of toroidal pores again relies on AMPs binding to lipids, but this time the association causes the bacterial membrane to bend inwards through the pore, retaining some association of the AMP-pore components with the lipid head groups of the bacterial membrane (e.g., the fish AMP piscidin from the striped bass, *Morone saxatilis* x *M. chrysops* [38]). The carpet mechanism operates through AMPs aggregating on the bacterial membrane and disrupting the bilayer in a detergent-like fashion, which leads to the formation of micelles (e.g., dermaseptin from the frog genus *Phyllomedusa* [35]). A few AMPs, such as the amphibian peptide buforin II, can translocate across the membrane without permeabilization and subsequently act on intracellular targets [39]. Otherwise there is very little known of the mechanisms of action of individual conventional AMPs from marine organisms and this is in urgent need of clarification.

After initial membrane disruption has taken place, killing may proceed by a number of ways. The most commonly assumed mechanism is direct cell lysis via membrane rupture, however, other possible mechanisms include inhibition of cell wall, protein or nucleic acid synthesis (e.g., tachyplesin from the horseshoe crab [40] and pleurocidin from the winter flounder, *Pleuronectes americanus* [41]), inhibition of enzyme activity (e.g., human histatins [42]), and binding to heat shock proteins and other chaperones (e.g., the bee AMP apidaecin [43]), which prevents the repair or elimination of misfolded and damaged proteins. The antimicrobial activity of AMPs has been quantified using a huge diversity of bacteria and a number of different methods. A standard way of expressing antimicrobial activity is the minimum inhibitory concentration (MIC), which is the lowest concentration of a peptide that inhibits the growth of bacteria after a predetermined incubation time, typically 24 hours. Other measures commonly used are the concentration that inhibits 50% of bacterial growth (IC_50_) and percentage growth inhibition. In the following sections, conventional AMPs from marine and aquatic animals are described according to the structural classification scheme in Table 2.

### 2.1. Linear, α-helical peptides

This peptide class comprises short, linear structures that have spatial segregation of hydrophobic and hydrophilic residues, with the peptides adopting an α-helical conformation when interacting with bacterial membranes. These peptides are extremely widespread throughout non-marine animal taxa and were first recorded in amphibians (magainins) and insects (cecropins) but amongst marine and aquatic animals their distribution to date appears mainly among ascidians and fish (Table 2). However, the peptide homarin, identified in the lobster, *Homarus americanus*, has sequence similarity with the short, linear α-helical temporins from the European red frog, *Rana temporaria*, but further information is needed to confirm the secondary structure of homarin [15].

#### 2.1.1. Invertebrates

The α-helical ascidian peptides include the clavanins and styelins, both groups purified from the haemocytes of *Styela clava* [25,30]. The four native clavanins are 23-residue, 2.6 kDa AMPs that possess rapid, broad-spectrum antimicrobial activities, inhibiting the growth of Gram-negative (*Escherichia coli*) and Gram-positive (*Listeria monocytogenes*) bacteria, as well as the fungus, *Candida albicans* [25]. Synthetic clavanin A is bactericidal against *E. coli* and *L. monocytogenes* at <4 μg mL^−1^ within 5 minutes of incubation. Clavanins are unusual in that they are histidine-rich and synthetic versions show different modes of antimicrobial activity at different pH values with greater potency under mildly acidic (5.5) pH compared with neutral pH [44]. At pH 7.0 killing is dependent on non-specific membrane disruption but at lower pH the histidines become protonated and membrane disruption probably occurs through interaction with the proteins responsible for generating ion gradients [45]. Clavanins retain their antimicrobial activity at high NaCl concentrations [44].

Styelins were also isolated from *S. clava* and comprise five 3.7 kDa phenylalanine-rich peptides [30,46]. Two were purified directly from the haemocytes [30] with an additional three members identified by molecular methodologies [46]. One of these, styelin D, was subjected to detailed analyses which reveal that it has potent activity against Gram-positive bacteria (MICs ~5–7 μg mL^−1^) at pH values from 5.5–7.4 [47]. It is also active against Gram-negative bacteria at pH 5.5 (MIC 2.1 μg mL^−1^) [47]. Styelins are active at high salt concentrations and the characterization of styelin D reveals that it contains an unusually high number of post-translationally modified residues that are thought to facilitate the antimicrobial activity in high salt and acidic pH conditions [47].

Other ascidian AMPs that form α-helical structures include dicynthaurin, a peptide from *Halocynthia aurantium* that forms a homodimer consisting of two, 30-residue monomers and has a single cysteine residue [26]. Unusually for an AMP purified from a marine animal, and in contrast to the clavanins and styelins, dicynthaurin is more potently antimicrobial at low salt concentrations, which indicates that the peptide might be compartmentalised in the cell [26]. A dimer structure also exists in halocidin from *Halocynthia papillosa*, although the peptide is a heterodimer consisting of one 18- and one 15-residue monomer linked by a disulphide bond [27]. Recently, two new AMPs, halocynthin and papillosin, have been isolated from *H. papillosa* [29]. Both of these AMPs are active against Gram-positive and Gram-negative bacteria [29].

#### 2.1.2. Fish

Alpha-helical amphipathic peptides are very common in fish as recently reviewed by Smith and Fernandes [31]. The first fish family of AMPs to be discovered was the α-helical pardaxins. These were isolated from the skin glands of Red Sea Moses sole, *Pardachirus marmoratus*, on the basis of their cytotoxic, pore-forming activities [48,49]. Pardaxins were originally described as toxins with anti-predatory function but subsequently they have been found to be active against Gram-positive and Gram-negative bacteria [50].

However, most fish α-helical peptides are members of the piscidin family, which includes the pleurocidins and piscidins [31]. Pleurocidins are 25-residue peptides first isolated from the skin mucus of winter flounder, *Pleuronectes americanus*. [51]. They have broad-spectrum antimicrobial activities [51] and inhibit DNA, RNA and protein syntheses [41]. Piscidins are 22-residue peptides first purified from skin and gills of hybrid striped bass (*M. saxatilis* x *M. chrysops*) [52] and now known to be present in other Perciformes [53]. Also within the piscidin family are dicentracin from the European bass, *Dicentrarchus labrax* [54], chrysophsins from red sea bream, *Chrysophrys major* [55] and epinecidin from the orange-spotted grouper, *Epinephelus coioides* [56]. All piscidins show broad-spectrum antimicrobial activity, probably killing cells via toroidal-pore formation [38,57–59]. Lee *et al.* [60] determined the solution structure of piscidin-1 and established that it is the conformational flexibility at the boundary between the hydrophobic and hydrophilic regions that is the critical factor for membrane selectivity and antibacterial activity. Recently, piscidin-2 has been found to cause cell membrane damage to three fungal strains known to cause infections in humans [61]. Importantly, they are expressed in mast cells [57], granules of acidophilic phagocytes and in gill, skin, stomach and intestinal epithelia [53]. In gilthead seabream, they are stored in the granules of the phagocytes and are delivered to the phagosome following uptake of bacteria by these cells [53]. Although attractive as potential candidates for topical application use because of their activity at high salt concentrations [52], the disadvantage of piscidins in this respect is their haemolytic and cytotoxic properties [57].

### 2.2. Cysteine-rich peptides

This group of conventional AMPs contains representatives from bivalve molluscs, decapod crustaceans, horseshoe crabs and fish (Table 2). The most prominent group of cysteine-rich AMPs is the defensins. These are defined as peptides with characteristic β-sheets and a precise arrangement of 6 cysteines forming three disulphide bonds [62]. However, with the increasing rate of discovery of new AMPs from invertebrates, there are a number of peptides which do not fit the standard defensin definition (e.g., the MGD-1 peptide from *Mytilus galloprovincialis*; [63]), yet share the cysteine-rich, β-sheet or disulphide bond features of the group. Such peptides also include the large family of crustins, which are cysteine-rich (containing eight cysteines) yet are characterized by the possession of a 4-disulphide core, rather than classical β-sheet arrangements with six cysteines [14].

#### 2.2.1. Invertebrates

The first groups of cysteine-rich AMPs to be purified from invertebrates are the tachyplesins, polyphemusins, tachystatins, big defensin and tachycitin, all from the horseshoe crabs, *Tachypleus tridentatus* or *Limulus polyphemus*, as reviewed by Iwanaga [64]. Features shared by these peptides include broad-spectrum antimicrobial activities, strong amphipathicities, the presence of β-sheets and β-turns and two or more disulphide bonds [64]. The tachyplesins are 17-residue AMPs that contain an anti-parallel β-sheet linked by a β-turn, and two disulphide bonds [21,64]. The tachyplesins bind lipopolysaccharide (LPS) [21] and target the bacterial inner membrane by altering potassium permeability [65]. Their MICs range from 0.8–12.5 μg mL^−1^ against susceptible bacteria and fungi [21]. The polyphemusins are 18-residue peptides that share with tachyplesins both β-sheets, β-turn and two disulphide bonds [21]. Polyphemusins have antimicrobial activity spectra that are similar to the tachyplesins, with MICs ranging from 3.1–12.5 μg mL^−1^ [21].

The tachystatins of horseshoe crabs are AMPs that bind chitin, possess 3 disulphide bridges and a 3-stranded β-sheet, stabilized by cysteine residues [66]. The tachystatins share structural similarity with ω-agatoxins (venoms isolated from the funnel web spider) that also bind chitin and have weak antifungal properties [66]. The antifungal activities of tachystatins and ω-agatoxins are mediated by their chitin-binding properties but the weaker activities of ω-agatoxins (IC_50_ values of 0.5 and 7.8 μg mL^−1^, respectively) probably arise from the lack of strong amphipathic conformation [66]. Another horseshoe crab AMP that binds chitin is tachycitin, a 73-residue AMP containing five disulphide bonds [67]. The structure of tachycitin includes a β-hairpin loop with a 2-stranded β-sheet, and shares some sequence similarity to the hevein domain (a domain that is characteristic of peptides which bind chitin and have antimicrobial activities [67]).

The big defensin of horseshoe crabs has 79 amino acid residues and three disulphide bonds with the disulphide motif identical to the β-defensins of bovine neutrophils [20]. The N- and C-termini of the big defensin show differential antimicrobial activities and, while the N-terminus is highly active against Gram-positive bacteria, the C-terminus is most potent against Gram-negative species [20]. The big defensin has an unusual structure, containing three α-helices, a double-stranded parallel β-sheet at the N-terminus and a four-stranded anti-parallel sheet at the C-terminus, thus creating two distinct domains [68]. The N-terminus domain has a β1-α1-α2-β2 fold and contains a hydrophobic core, while the C-terminus comprises a β3-β4-β5-α3-β6 domain that adopts a compact structure held by its disulphide bonds [68]. The differential antimicrobial activities of the N- and C-termini are thought to arise from proteolytic cleavage by microbial proteinases, since cleavage with trypsin liberates the hydrophobic N and the cationic C-terminus fragments [68]. Recently, a big defensin gene (*AiBD*) has been cloned from the bay scallop, *Argopecten irradians* [69]. The deduced amino acid sequence of this gene shares 48% identity with the horseshoe crab big defensin [69]. The arrangements and spacings of the cysteine residues are highly conserved between these two AMPs, while a recombinant AiBD protein has a similar spectrum of antimicrobial activity to the horseshoe crab peptide [69].

Among the invertebrate cysteine-rich AMPs are also the defensins, myticins, mytilins and mytimycin, purified from the haemocytes of marine mussels belonging to the genus, *Mytilus (M. edulis* and *M. galloprovincialis)*. These AMPs have been subjected to the most detailed investigations of all bivalve peptides reported to date [10]. All the *Mytilus* spp. AMPs are amphipathic and have hydrophobic and cationic features, although there are variations in their structures and activities [10]. Whereas the *M. edulis* defensin has six cysteines like the arthropod defensins [70], the defensin purified from *M. galloprovincialis* (MGD-1) has eight cysteines arranged in four disulphide bonds [63]. MGD-1 is principally active against Gram-positive bacteria [71] and contains the cysteine-stabilized α-β motif (CSαβ; [72]). Its antimicrobial activity is dependent on the β-hairpin loop that is involved in binding, growth inhibition and bacterial membrane permeabilization [71]. In contrast to the insect defensins, neither the defensin nor mytilin B from *M. galloprovincialis* appear to be inducible, as transcription appears to decrease with bacterial challenge, although plasma levels of the peptide increase [73,74].

The peptides in the myticin family are structurally similar to the *Mytilus* spp. defensins in that they have eight cysteine residues, comprising four disulphide bonds, and are potently active against Gram-positive bacteria [75]. The mytilin peptides exhibit diverse antimicrobial spectra depending on their different isoforms. Of the five mytilin isoforms so far purified from *Mytilus* spp., three (B, C and D) are active against Gram-positive and Gram-negative bacteria, one (G1) is active only against Gram-positive strains, while two (B and D) are also antifungal [74]. Recently the full structure of synthetic mytilin was found to share similarities with MGD-1 as both peptides contain the CSαβ motif, although the N-terminus section of mytilin is shorter and the β-hairpin more extensive in the mytilin peptide [76]. The antifungal peptide mytimycin from *M. edulis* has received less attention compared with the other *Mytilus* spp. AMPs, but is ~6.5 kDa and is thought to contain 12 cysteines [70]. In comparison with other marine invertebrates, *Mytilus* spp. AMPs have been the best studied with respect to their localization in cells and tissues, their expression under different conditions [77] and during their life-history [78], their differential involvement in antimicrobial defence [79] and the distribution of different isoforms in individual animals [80].

Defensins have also been purified from the gills of the American oyster, *Crassostrea virginica* [81] and identified at the molecular level from the mantle tissue and haemocytes of the Pacific oyster, *Crassostrea gigas* [32,33]. Like the *Mytilus* spp. defensins, the defensins from *C. gigas* have eight cysteines, the CSαβ motif and are principally active against Gram-positive bacteria [32]. The 38-residue defensin from *C. virginica* has high sequence homology (62%–73%) with the arthropod defensins, has six cysteines and is active against both Gram-positive and Gram-negative bacteria [81]. A few AMPs have been reported from clams, scallops and abalone, but as most of these have arisen from molecular methodologies relatively little is known about the features or activities of either native or recombinant peptides [10].

The two main families of cysteine-rich AMPs from crustaceans are the crustins [14] and penaeidins [17]. Carcinin, the first crustin to be discovered, was purified from the shore crab, *Carcinus maenas* [82], however it was not designated a crustin until much later [83]. Crustins have been found in every decapod crustacean studied, with gene sequences similar to crustins also present in the amphipod, *Gammarus pulex*, the brine shrimp, *Artemia salina*, and the copepod, *Calanus finmarchius* as discussed by Smith *et al*. [14]. Although first identified by their antimicrobial activities, the crustins are not especially potent nor do they exhibit broad-spectra antimicrobial activities, as they tend to affect mainly Gram-positive bacteria. Smith *et al.* [14] define crustins as cationic, cysteine-rich antibacterial polypeptides, ~7–14 kDa, containing one whey four-disulphide core domain (WFDC; also known as a WAP domain), a conserved structure of eight cysteines forming a 4-disulphide core at the C-terminus. In addition, Smith *et al.* [14] propose that three types of crustin (I, II, III) exist, each distinguished by the domain structure between the signal sequences and the C-terminus domain containing the WFDC. All three types possess a signal sequence at the N-terminus and the WFDC domain at the C-terminus [14]. The WFDC domain is tightly constrained by three disulphide bonds and has a small α-helix that probably accounts for the antibacterial effect of the crustins. As well as the WFDC domain, Type I crustins also possess a cysteine-rich domain that contains six cysteines [14]. Type II crustins have yet another domain, rich in glycines between the signal sequence and the six cysteine-region [14]. Type III crustins have neither the glycine- nor cysteine-rich domains [14]. It has been suggested that the crustins could have other roles in the normal physiology of the animals, as carcinin expression can change with varying environmental factors and with the moult cycle of the crab, yet shows little change in expression when subject to bacterial challenge [14].

The penaeidins, first identified from the shrimp *Litopenaeus vannamei*, [84] have now been isolated from a number of shrimp species [17]. Their distribution appears to be confined to shrimp unlike the crustins, which are present in shrimp as well as all other decapods [14]. Penaeidins are 5–6 kDa and, in addition to the signal sequence, they comprise two domains: a proline-arginine rich N-terminus and a cysteine-rich (typically six cysteine residues) C-terminus. Of the two domains, the cysteine-rich one is more compact and includes an α-helix, stabilized by three disulphide bonds. Five families of penaeidins have been classified to date, with their spectra of activities predominantly against Gram-positive bacteria and fungi [84]. Penaeidins have chitin-binding activity, which is also attributed to the C-terminus region, but the presence of a specific domain, such as a hevein domain, has not been confirmed [85].

A few cysteine-rich peptides have been purified from other invertebrate groups, e.g., aurelin from the jellyfish, *Aurelia aurita* [6]. Aurelin contains six cysteines forming three disulphide bridges and comprises a signal peptide, anionic propiece and a mature cationic part, thus resembling certain structural features of the defensins [6]. Unlike the defensins the distribution of cysteine residues is more reminiscent of the potassium-blocking channels of sea anemone toxins, although such a property in aurelin has yet to be investigated [6]. Aurelin is active against Gram-positive and Gram-negative bacteria, with MICs ranging from 7–22 μg mL^−1^ [6].

The first AMPs to be isolated from any echinoderm are the strongylocins from the sea urchin, *Strongylocentrotus droebachiensis* [24]. These are cationic cysteine-rich peptides (5.6 and 5.8 kDa) that contain six cysteines likely to form three disulphide bonds, although the cysteine arrangement patterns differ from those observed in other AMPs with six cysteines [24]. Strongylocins exert antimicrobial activities against both Gram-positive and Gram-negative bacteria (IC_50_ ranging from 1.3–5 μM) and they contain bromotryptophan; a feature they share with some other AMPs from marine sources [24] (e.g., hedistin [8]).

#### 2.2.2. Fish

Amongst the cysteine-rich AMPs in teleost fish are three families: cathelicidins, defensins and LEAPs [31]. By screening cDNA libraries, putative cathelicidins have been found from rainbow trout, *Oncorhynchus mykiss* [86] and an EST database of Atlantic salmon, *Salmo salar* [87]. Using molecular methodology, further cathelicidins have been identified for Arctic char, *Salvelinus alpines*, Atlantic cod, *Gadus morhua*, and brook trout, *Salvelinus fontinalis* [88]. Moreover, cathelicidin genes have been reported for jawless fish, namely the Atlantic hagfish, *Myxine glutinosa* [89]. The structures of teleost cathelicidins have some features in common with mammalian cathelicidins, e.g., the presence of four cysteines at the C-terminus regions forming two disulfide bonds [87]. However, in general, the signal peptides of teleost cathelicidins tend to have fewer amino acid residues but a longer cathelin-like domain than mammals [87]. Little information is available with respect to the native peptides but synthetic rainbow trout cathelicidins are active against Gram-positive and Gram-negative bacteria [87].

Defensins from teleost fish, as with cathelicidins, have been identified by molecular methodologies rather than purification of the native peptides. Zhou *et al.* [90] used EST and complete genome data to identify defensins from zebrafish and pufferfish that resemble the β-defensins of birds and mammals. The fish defensins contain six conserved cysteines in the region of the mature peptide and three β-strands, although one difference to the avian and mammalian defensins is the presence of an extra helix in one of the zebrafish peptides [90]. Falco *et al*. [91], using a recombinant protein based on rainbow trout defensin, found it to be antiviral against viral haemorrhagic septicaemia rhabdovirus (VHSV), one of the most troublesome diseases in fish aquaculture. More recent studies have cloned three novel β-defensins from rainbow trout, all of which appear to be constitutively expressed but increase in expression during bacterial and simulated viral challenges [92]. Similarly, a β-defensin-like gene from the olive flounder, *Paralichtyus olivaceus*, has been identified, which is expressed in larval fish just one day after hatching, although the expression declines between 1–35 days post-hatching [93]. Moreover, β-defensin expression in juvenile fish is induced under conditions of bacterial challenge and the recombinant peptide suppresses the growth of *E. coli* [93].

The last major group of cysteine-rich AMPs from fish is the LEAPs (liver-expressed antimicrobial peptides) [31], the acronym reflecting the original identification of the peptide family in the human liver [94]. Peptides belonging to the LEAP family include hepcidins from several species (e.g., winter flounder, turbot and red sea bream), Sal-1 and Sal-2 from Atlantic salmon, JF-1 and JF-2 from Japenese flounder, and LEAP-2 from catfish and trout (reviewed by Smith and Fernandes [31]). The first fish LEAP to be identified, the hepcidin from the gills of striped bass [95], has a similar structure to human hepcidin and consists of two anti-parallel β-sheets and eight cysteines forming four disulphide bonds [96]. However, two LEAPs have since been found that only possess two disulphide bonds [31]. Their activity spectra have been determined mainly using synthetic peptides, which show activity against Gram-negative bacteria and fungi [96]. Under conditions of bacterial challenge, the expression of hybrid bass hepcidin is detectable in most tissues, but is highly up-regulated in the liver (>4,000 fold) [95]. Some fish LEAPs, e.g., those from catfish and turbot, are expressed very early in the life cycle in comparison with other fish AMPs and their involvement in iron regulation has also been suggested [31].

### 2.3. Cationic peptides, amino acid enriched

None of the known fish AMPs are classified as amino acid enriched cationic peptides [31] (Table 2). Cationic, amino acid enriched peptides from marine invertebrates include the proline-rich peptide, Bac-like, isolated from the shore crab, *Carcinus maenas* [12] and the partial sequence of a proline-rich peptide (callinectin) from the blue crab, *Callinectes sapidus* [13]. In addition, molecular techniques have enabled the identification of a proline-rich peptide (*Cg*-prp) from the Pacific oyster, *C. gigas* [97] (Table 2). The Bac-like peptide from *C. maenas* has activity against both Gram-positive and Gram-negative bacteria and has some functional and partial sequence similarity to bovine cathelicidin AMPs e.g., bactenecin-7 [12]. The callinectin AMP is active against *E. coli* and has a hydrophobic region but the proline residues are arranged differently compared with existing proline-rich AMP sequences [13]. *Cg*-prp was originally identified from EST data but synthetic fragments show weak antimicrobial activites [97].

Further examples of amino acid enriched AMPs from marine invertebrates include aracin and hyastatin, from the spider crab, *Hyas arenaeus*, both of which contain more than one distinct domain. Aracin is a 37-residue AMP, which has an N-terminus enriched for proline and arginine and a C-terminus containing four cysteines forming two disulphide bridges [11]. The native peptide is active against both Gram-positive and Gram-negative species, with IC_50_ values ranging from 0.8–12.5 μM [11] (Table 2). Hyastatin, an 11.7 kDa glycine-rich AMP, possesses a domain at the C-terminus containing six cysteines arranged in a similar three disulphide bond formation to that of the penaeidins [16]. This AMP is active against Gram-positive and Gram-negative bacteria as well as fungi, with IC_50_ values ranging from 0.4–12.5 μM [16] (Table 2). Activity against Gram-positive bacteria is severely reduced in the recombinant peptide by removal of the cysteine region [16]. Both native hyastatin and its N-terminus region can bind chitin, which may facilitate its antifungal capability or suggest a more multi-functional role in the animal [16].

### 2.4. Miscellaneous AMPs

Three novel AMPs have been purified from marine worms. Two isoforms of the AMP, arenicin, have been purified from the immune cells of the lugworm, *Arenicola marina* [7]. The arenicins are 21-residue peptides containing 2-stranded β-sheets as well as one disulphide bridge forming an 18-residue ring [7]. They are regarded as a new class of AMP largely because of the ring structure [7]. They possess strongly hydrophobic regions separated by positively charged arginine side chains and the presence of arginine is thought to contribute to high salt tolerance [98]. Arenicins completely kill *E. coli* within 5 minutes at a concentration of 5 μM probably by membrane permeabilization [98] (Table 2). A second annelid peptide, named perinerin, has been purified from the clamworm, *Perinereis aibuhitensis* [9]. Perinerin contains four cysteine residues forming two disulphide bridges with the most abundant amino acid being arginine [9]. This peptide was not classed as being highly enriched for any amino acid and despite the presence of cysteine residues and disulphide bridges, its average percentage identity to other cysteine-rich AMPs was less than 30% [9]. Perinerin is active against Gram-positive (lowest MIC is 1.5 μg mL^−1^) and Gram-negative (lowest MIC is 3.1 μg mL^−1^) bacteria, as well as the fungus, *Paecilomyces heliothis* (MIC 12.5 μg mL^−1^) [9] (Table 2).

Finally, the most recent AMP to be isolated from a marine annelid is hedistin purified from the ragworm, *Nereis diversicolor* [8]. It shares no significant similarity with other AMPs [8]. Both native and synthetic hedistins are active against Gram-positive and Gram-negative bacteria with MICs as low as 0.4–0.8 μM (Table 2). Hedistins are notable for containing bromotryptophan, although the synthetic version without bromines is still effective, indicating that bromination is not essential for activity [8].

## 3. Unconventional Anti-infectives

### 3.1. Antimicrobials derived from intracellular structures

#### 3.1.1. Histones

Histones are proteins present in the nuclei of all eukaryotic organisms. They are conspicuous proteins of chromatin and are responsible for the packaging of DNA, essentially serving to wind up the long DNA strands in a spool-like manner. There are several types of histone of which H2A, H2B, H3 and H4 are the core histones that form the nucleosome, and H1 and H5 are the linker histones. That histones have potent antimicrobial properties has been known for over 50 years [99] but at that time there was no theoretical concept as to how they might interact with bacteria for the host’s benefit, so little attention was paid to this discovery. By the late 1990s, histones were reported to contribute to the antibacterial activity of wound blister fluid [100]. Then reports began to emerge that histones account for a large proportion of the antibacterial activity of skin exudates from amphibians [101] and teleost fish [102–105]. Noga *et al.* [106] also found that histones are active against fish-parasitic dinoflagellates. Histones have now been reported to be present in the skin mucus of several fish taxa, including Salmoniformes, Siluriformes and Pleuronectiformes [31] (Table 3).

Amongst the core histones, H2A is a potent antibacterial agent. It is a 13.6 kDa protein able to kill Gram-positive bacteria at sub-micromolar (<0.4 μM) concentrations within 30 minutes *in vitro* [105]. It also has some weak activity against the yeast, *Saccharomyces cerevisiae*, but it is not haemolytic to trout erythrocytes at antimicrobial concentrations [105]. It does not appear to form stable ion channels in the bacterial cell membrane but reconstituting pure H2A in a planar lipid bilayer does disturb the membrane [105]. Interestingly, it is not only the intact H2A protein that has antimicrobial effects but also fragments generated from the N-terminus by proteolytic cleavage (Table 3). Such fragments include parasin-1 from the catfish, *Parasilurus asetu*, [101], hipposin from Atlantic halibut *Hippoglossus hippoglossus* [109], buforins from toads [114], and abhisin from abalone [110]. H2A or fragments derived from it have also been recorded for scallop, *Chlamys farreri* [115] and shrimp, *Litopenaeus vannamei* [108] (Table 3). The liberation of parasin-1 from H2A in fish mucus is mediated through the enzyme cathepsin D [116] with a second enzyme, matrix metalloproteinase 2, involved in the regulation of this process [117].

Core histone H2B also has antimicrobial activity, which was first noted for mouse macrophages [118] and subsequently reported for channel catfish skin exudates [102], epidermal secretions from Schlegel’s Green tree frog [119], surface mucus from Atlantic cod [111] and shrimp haemocytes [108] (Table 3). Histone H2B is active against the fish pathogens, *Aeromonas hydrophilia* and *Saprolegnia* [102]. Histone H4 is another microbicidal core histone, having been found to be one of the active factors in human sebocyte secretions [120]. H4 from shrimp haemocytes has antibacterial properties [108] but reports for its antimicrobial role in other marine or aquatic organisms are scant. With H3, synthetic H3–like peptides are antibacterial [121] and H3 is present in mucus extruded from the hagfish *Myxine glutinous* [122].

The linker histones also have anti-infective properties (Table 3). H1 exhibits antimicrobial properties and has been isolated from several species, including humans [123], mice [118], fish [104,107,124] and shrimp [108]. H1, when isolated from Coho salmon, is active against *E. coli* with a MIC of 31 μg mL^−1^ [104]. A 26-residue N-terminus fragment of H1 from Coho salmon is also active against various fish pathogens, including *Aeromonas salmonicida*, *Listonella anguillarum* and *Salmonella enteritica* [103]. In winter flounder, the expression of this protein is up-regulated following immune stimulation, which coincides with an increase in the antibacterial activities of serum and mucus [103], indicative of its role in systemic as well as mucosal response to non-self challenge. Significantly, the C-terminus from H1 (from *O. mykiss*) has been shown to generate a 7.2 kDa fragment, termed oncorhyncin II, that has very high potency (~10 times greater than cecropin P1) against both Gram-positive and Gram-negative bacteria, probably by destabilizing the bacterial membrane, although not necessarily by pore formation [107]. Therefore, H1 is strongly antibacterial not only as the complete molecule but through fragments at both the N and C-termini (Table 3).

Histones are highly conserved alkaline and water soluble proteins showing remarkable similarity across divergent phyla. For example, antisera to human histones are known to cross react with histones from several invertebrates [125]. It is therefore highly likely that histones from most, if not all eukaryotic species, will show similar potent antimicrobial activity as those described above, although the extent and modes of histone participation in host defence is, as yet, far from clear. Their possession of amphipathic secondary structures and microbicidal properties might be merely incidental and have no survival value. Hirsch’s finding in 1958 [99] of the antimicrobial properties of histones was disregarded as not physiologically relevant for many years but the presence of these peptides in skin secretions of fish is less surprising given that fish skin is living and not keratinized. The epithelial surface is constantly at risk of abrasion and sloughing, so damage to the cells from such minor injuries might permit histones and other intracellular antibacterial proteins, along with phospholipid-derived free fatty acids, to become exposed to epibionts or potential invaders from the surrounding water. Enzymes, such as cathepsin D, also expressed in fish epidermal mucosa, are now known to aid the release of histone fragments [116]. However, this does not explain how histones or other intracellular antibacterial factors might interact with infectious agents within the body tissues of any animal.

#### 3.1.2. Other intracellular proteins

Apart from the core and linker histones, research on rainbow trout has established that other intracellular proteins have potent antibacterial activities against infectious agents. One from fish is a 6–7 kDa N-terminus fragment of a high mobility non-histone chromosome protein, H6, which has very powerful activity against a range of bacteria (MICs 0.06–0.12 μM) [112]. This fragment, designated as oncorhyncin III, is salt sensitive, non-haemolytic and able to destabilize planar lipid membranes [112]. Another intracellular protein found to have antibacterial effects is a 6.6 kDa fragment derived from the 40S ribosomal protein, called S30 [113] (Table 3) from skin secretions of *O. mykiss*. S30 inhibits the growth of Gram-positive and Gram-negative bacteria but has strongest microbicidal activity against the Gram-positive species [113]. Three ribosomal-derived proteins and peptides, namely L40, L36A and L35, (6.4, 12.3 and 14. 2 kDa, respectively), have also been isolated from the epidermal mucus of Atlantic cod, *Gadus morhua* [111] (Table 3). The remarkably strong activity of core and linker histones, as well as ribosome-derived proteins, raises questions about the contribution of their anti-infective properties to defence, as they would normally not be exposed to invasive bacteria, even intracellular ones.

### 3.2. Membrane-derived antimicrobial compounds

#### 3.2.1. Free fatty acids

The cell membranes of eukaryotic organisms can also be an important source of several antimicrobial compounds, with free fatty acids (FFAs) particularly prominent. Fatty acids, especially unsaturated varieties, are rarely found in their free form inside living cells [126,127] but more usually they are bound to other groups, such as phosphates, sugars or glycerol, forming lipids. Phospholipids are the major structural components of cell membranes, while galactolipids are located in the chloroplasts and triglyercides may form cellular energy reserves as lipid stores. Fatty acids can be released from cell membranes as FFAs upon cell or tissue damage caused typically by a consumer or a pathogen [126–130].

There are numerous FFAs with antimicrobial effects and their spectra of action and potencies are influenced by the degree of saturation, length of carbon chain and the orientation of the double bonds [131] (Table 4). Briefly, amongst the saturated FFAs, capric acid (C10:0) and lauric acid (C12:0) tend to be the most active [132–134], while amongst the monounsaturated FFAs, myristoleic (C14:1) and palmitoleic acid (C16:1) often are the most potent [132,133] (Table 4). Monounsaturated FFAs with less than 14 or more than 16 carbon atoms in the carbon chain tend to have rather less activity [132,133] (Table 4). Many polyunsaturated FFAs are potently antibacterial, particularly those with 18 and 20 carbons in their carbon chain [132]. Interestingly, a direct relationship can exist between the number of double bonds in the carbon chain and the antibacterial activity of the FFA [132,135,136] (Table 4). In general FFAs with *cis*-orientated carbon-carbon double bonds have greater antimicrobial activities compared with those FFAs containing *trans*-orientated bonds [132,137] (Table 4).

The microbicidal activities of membrane-derived FFAs have been extensively studied and these seem to be important defence effectors, especially in the macro- and micro-algae [126,128–131]. Micro-algae, even those species with partial or complete cell armouring, such as diatoms, can lose structural integrity through mechanical damage by consumers, osmotic shock, water turbulence and viral- or bacterial-induced lysis. Such damage may result in the release of high concentrations of FFAs, particularly from the phospholipids of the cell membrane and the galactolipids of the chloroplasts, into the vicinity of the damaged cell [127,138,139]. The liberation of FFAs is immediate and is carried out by a family of hydrolytic enzymes called lipases [127,140]. During pathogen-induced lysis the bioactive FFAs are thus brought into close contact with any adjacent prokaryotes, whether they are the same pathogen that may have initiated the damage in the first instance, or are other heterotrophic opportunists. Killing of these microbes by the released FFA will not, of course, save the damaged micro-alga but could help protect its neighbours and would be beneficial, in evolutionary terms, to the population if these surrounding cells are clonal or close genetic relatives. In this way, pathogen transmission within a micro-algal population can be brought under some control.

The production of FFAs is a multi-faceted defence strategy in the micro-algae, as these compounds are toxic to many threats to host survival, including viruses, protozoans and consumers [126,141]. Further, this defence strategy can be considered metabolically inexpensive, as the FFAs come from vital structures within the cell and the lipases involved may pre-exist to serve alternative essential functions in living cells [142,143]. While micro-alga-pathogen interactions are not well characterised there is a growing literature in this field [144–147] but future experimentation must confirm the precise role for FFAs against recognised micro-algal pathogens. Pathogen-damaged tissues of macro-algae produce FFAs in a similar manner to the micro-algae and these not only kill the pathogen and prevent its spread through the host but the FFAs can also act as signals (or precursors of signalling molecules) that trigger downstream systemic defence responses [128–130].

The marine diatom *Phaeodactylum tricornutum* is a popular model for investigations of micro-algal physiology and it is a good example of a micro-alga that forms antibacterial FFAs through lipase action after mechanical disruption of the cells [148]. The main ones are medium- and long-chain unsaturated varieties, particularly eicosapaentaenoic acid (C20:5 n-3) [149], hexadecatrienoic acid (C16:3 n-4) and palmitoleic acid (C16:1 n-7) [150]. These unsaturated FFAs are typical of those isolated from extracts in similar antibacterial bioassay-guided fractionation studies of other macro- and micro-algal species [151,152]. Eicosapaentaenoic acid (EPA) has strong activity against a wide range of Gram-positive and Gram-negative marine and non-marine bacteria *in vitro*, including *Pseudomonas aeruginosa* and methicillin-resistant *Staphylococcus aureus* (MRSA) [149] (Table 5).

Fish and shellfish pathogens, such as *Lactococcus garviae*, harmful Vibrios and *L. anguillarum* are also killed by EPA although it does not appear to affect fungi [149,153] (Table 5). Similarly, hexadecatrienoic acid (HTA) isolated from *P. tricornutum* displays activity against the Gram-positive pathogen, *S. aureus* [150]. Palmitoleic acid (PA) is active against various non-marine Gram-positive human pathogens at micromolar concentrations and begins to kill bacteria upon immediate exposure [150]. Like EPA, HTA appears to have little or no activity against fungi [150], although there are reports of antifungal activity attributable to PA [154]. Interestingly, higher levels of these and other bioactive FFAs are present in the fusiform morphotype of *P. tricornutum* (the morphotype that tends to dominate in the plankton) compared with the oval morphs of this micro-alga, which tend to occur on surfaces [155]. It is likely that this is a functional adaptation to prevailing conditions but shows that yields of antibacterial FFAs, both naturally in the sea and experimentally in the laboratory, can be influenced by growth and environmental conditions. MICs for FFAs against susceptible bacteria are typically 100 μM and greater [133,156–158] but, in certain cases, they can be as much as one order of magnitude more potent [133,135,159]. However, whilst FFAs may not be as potent as AMPs by direct comparison, their fast accumulation and thus potentially greater local concentrations mean that they can attain the necessary concentrations to exert their antimicrobial activities.

FFAs can be bactericidal (kill ≥99.9% of original inoculum) or can reversibly inhibit bacterial growth without complete killing (bacteriostatis) [160], although the mechanisms by which they act have yet to be fully characterised. There are several ways that they are thought to attack bacterial cells but, of these, the cell membrane is probably the prime target [131]. Likely cell processes targeted by FFAs include interference with cellular energy production by disrupting the electron transport chain and oxidative phosphorylation, inhibition of enzyme activity, impairment of nutrient uptake, generation of toxic peroxidation and auto-oxidation degradation products or direct membrane disruption causing bacterial cell lysis [131,161]. The exact process responsible is likely to depend on the bacterial strain, the FFA concerned and its concentration. It is also probable that FFAs work simultaneously on multiple targets within the bacterial cell, thus reducing the likelihood of inducible bacterial resistance.

#### 3.2.2. Oxylipins

In many species of macro- and micro-algae, the FFAs released by lipases from the phospholipids and galactolipids of damaged cells are very rapidly transformed into other compounds [127,130,138,139,162]. The usual process is for the FFA to be oxygenated initially by lipoxygenase enzymes to give intermediary hydroperoxides [163,164]. Further enzymes convert the hydroperoxides into oxylipins, of which there is a great variety, including unsaturated aldehydes and hydroxyl-, keto- and epoxyhydroxy fatty acid derivatives [165–168]. The exact repertoire of FFA-derived oxylipins depends on the algal species (and sometimes the strain) and the particular suite of enzymes that they express [169].

Importantly, the oxylipins produced may have appreciably more or, in some cases, less antibacterial activity than the FFA from which it was derived [136,170]. Amongst the micro-algal-derived oxylipins, it is the antibacterial activities of the polyunsaturated aldehydes (PUAs) that have attracted most recent attention. These PUAs are generated by diatoms including *Skeletonema costatum* and *Thalassiosira rotula* [169]. Diatom-derived PUAs are produced from C16, C20 and C22 polyunsaturated FFAs, particularly HTA, hexadecatetraenoic acid (C16:4 n-1) and EPA [138,139,162–164]. These FFAs are first oxygenated by a lipoxygenase and then hydroperoxide lyases act on the hydroperoxide intermediates to give PUAs, including heptadienal (C7:2 n-3), octadienal (C8:2 n-4), octatrienal (C8:3 n-1) and decadienal (C10:2 n-3) [138,139,162–164]. One typical, well-studied and highly antibacterial PUA is decadienal (DD), which is probably derived from the polyunsaturated fatty acid, arachidonic acid (C20:4 n-3) [127,141]. DD exhibits strong activity against important Gram-positive and Gram-negative human pathogens, such as MRSA and *Haemophilus influenzae* with MIC values of 7.8 and 1.9 μg mL^−1^, respectively [171]. DD at micromolar concentrations also detrimentally affects the growth of diverse Gram-positive and Gram-negative marine bacteria [172,173] (Table 5). Indeed, DD is also highly antagonistic towards numerous non-marine bacterial species (Table 5) and there is also evidence for activity against fungi [172]. Little work has been performed to characterise the antibacterial activities of PUAs and further studies in this field are warranted. Importantly, it is necessary to discriminate between bactericidal and bacteristatic activities. Such studies would begin to elucidate their specific mechanism(s) of antibacterial action.

### 3.3. Pigment or pigment-derived antimicrobials

Many marine or aquatic organisms synthesize pigments for a variety of purposes, including camouflage or warning colouration, oxygen transport, signalling, or protection against ultraviolet radiation, predation and microbial colonization. Some of these pigments serve multiple functions. Blood borne pigments are especially well placed to contribute to antibiosis as they permeate all tissues and organs. It is therefore not surprising that a number of respiratory or other blood pigments either have direct antimicrobial properties or contain motifs that upon liberation by degradative enzymes are microbicidal (Table 6).

#### 3.3.1. Respiratory pigments

The antimicrobial properties of mammalian haemoglobin have been known for a long time [206] and the activities are now attributed to peptides derived from the α- and β-subunits within the haemoglobin tetramer [207,208]. With fish, Fernandes and Smith [174] have reported the partial purification by RP-HPLC of two cationic proteinaceous factors from the acid soluble erythrocyte extracts of *O. mykiss* (Table 6). One of these factors is active against Gram-positive and Gram-negative bacteria with MIC values of 7–14 μg mL^−1^ and 14–28 μg mL^−1^, respectively, while the other is active against the Gram-positive bacterium, *Planococcus citreus*, with a MIC of 1–2 μg mL^−1^ [174] (Table 6). All of these values are within the sub-micromolar range and are approximately in the same order of magnitude as cecropin P1 but, as yet, the exact nature of these proteins is unknown [174]. These antimicrobial compounds could be haemoglobin-derived peptides but, as fish have nucleated erythrocytes, it is also possible that one or more of the activities might be due to histones or other intracellular structures, such as those described above. Other studies have established that two peptides derived from the β-subunit of catfish (*Ictalurus punctata*) haemoglobin have antibacterial activity against Gram-negative bacteria and these peptides can also kill *Ichthyophthirius multifiliis*, a ciliate parasite of this fish [209] (Table 6). As the transcript for at least one of these β-subunit-derived antimicrobial peptides from haemoglobin seems to be up-regulated in the skin and gill epithelium of *I. punctata* following *I. multifiliis* infection, it likely that these peptides might act in an anti-infective manner for the host [209].

Haemocyanin is another important oxygen carrying pigment in the blood of many invertebrates, especially large active ones, such as decapod crustaceans. Several studies have revealed its involvement in an anti-infective capacity (Table 6). With shrimp, intact haemocyanin can not only directly agglutinate bacterial cells [210] thereby restricting their ability to divide and spread around the body, but it also has antiviral effects [211].

Perhaps more interestingly, two antifungal peptides of 2.7 and 7.0–8.3 kDa, respectively are generated by limited proteolytic cleavage at the C-terminus of haemocyanin from the shrimp, *Litopenaeus vannamei* [175]. These peptides have strong activities against a range of fungi with MIC values of ~3–12 μM [175] (Table 6). They probably serve to kill fungi that may penetrate into the haemocoel through the carapace but their effects do not appear to be due to heavy metal or binding sites for bivalent ions [175]. In addition to these, another antibacterial peptide, astacidin-1, appears to be generated from the C-terminus of haemocyanin from the crayfish, *Pacifastacus leniusculus*, by proteolytic cleavage in acidic conditions [212]. This peptide is active against a range of Gram-positive and Gram-negative bacteria, including some human pathogens, at MIC values ranging from 2–20 μM [212]. The bioactivity of astacidin-1, which contains a β-sheet like many conventional AMPs, seems to reside at its N-terminus and its importance in host defence is demonstrated by its increased release into the haemolymph after crayfish have received injections of bacterial LPS [212].

#### 3.3.2. Other pigments

An example of a non-respiratory blood pigment that has antimicrobial effects is echinochrome A from echinoderms, particularly sea urchins (Table 6). Echinochrome is an orange-red coloured naphthoquinone (6-ethyl-2,3,5,7,8-pentahydroxy-1,4-naphthoquinone) that has iron chelating and free radical scavenging properties [213]. Echinochrome A is abundant in the red spherule coelomocytes of echinoid coelomic fluid [214]. It was found to have bactericidal properties by Service and Wardlaw [176] working with the common European sea urchin, *Echinus esculentus*. In this species it is present in the cells at 3–60 μg mL^−1^ and, importantly, it is active against a range of Gram-positive and Gram-negative bacteria at 50 μg mL^−1^ [176] (Table 6). It is highly likely to owe its antibacterial effects to iron-chelation and, as it is also present in urchin eggs and larvae, it may assist in preventing microbial colonization after spawning and fertilization.

Melanin is a pigment that occurs in many organisms: animals, plants, fungi and bacteria. Indeed, for many marine protostome invertebrates, particularly crustaceans and molluscs, and to a lesser extent, in annelids and ascidians, melanisation is a conspicuous immune response to infection. In these animals, wounds and abrasions to the external body surface rapidly become melanised, while internally melanin deposition accompanies the formation of haemocyte capsules around fungi, parasites or bacteria that have gained access to the haemocoel or coelomic cavity. The occurrence of melanin at sites of injury or infection in so many taxa has led to the general conception that melanin, and/or its quinone precursors, have anti-infective properties (Table 6) and a number of papers have appeared offering experimental data to support this [177–180]. However, it has also been argued that melanin is not a significant anti-infective, particularly as some fungi and pathogenic bacteria defend themselves against attack by the host immune system by utilizing melanin, either synthesized by themselves or produced by the host [215]. Certainly melanin has anti-oxidant properties, particularly through mopping up H_2_O_2_, so it may be exploited by some pathogens for protection against the effects of reactive oxygen intermediates generated by the host phagocytes. However other invaders, such as fungal hyphae, are essentially sequestered by this insoluble polymer into the haemocyte capsule matrix and then asphyxiated.

What may be important in relation to any anti-septic benefits of melanisation, at least in some protostome invertebrates, is the enzymatic oxidation of phenolic molecules to form the quinone precursors of melanin. The enzyme responsible is phenoloxidase (PO) and the activation of PO from its inactive precursor, proPO, is known to be a key defence response to microbial infection in many marine and non-marine invertebrates. Certainly, purified phenoloxidase from blood cells of the solitary ascidian, *Halocynthia roretzi*, has antibacterial activity in the presence of -(3,4-dihydroxy)-phenylalanine (L-dopa) [182] and indirect evidence has been provided that PO from the cephalochordate, *Amphioxus belcheri*, has antibacterial effects [183] (Table 6). In arthropods, phenoloxidase-catalyzed reactions have been found to produce antimicrobial reactive intermediates from natural substrates [216] and in shrimp silencing of the gene encoding prophenoloxidase by RNA interference results in a significant increase in bacterial load in the haemolymph [217]. Importantly, too, work on crustaceans has shown that proteolytic cleavage of haemocyanin, not only generates an antibacterial peptide from the C-terminus [212] but also produces PO from the N-terminus of subunit 2 [218]. Thus there is a clear link between haemocyanin, melanization reactions and microbial killing in invertebrates.

Not all pigments that show anti-infective effects are present in blood or body fluids circulating freely through the body. Indeed, the ink produced by some cepalopods and opistobranch gastropods upon attack or threat as defensive, anti-predator shields has been found to possess a number of factors with antibacterial, antifungal and/or cytotoxic activities [184,219,220]. At least one such pigment in the ink of octopus is melanin [181] but in gastropods, especially those belonging to the genera *Aplysia* and *Dolabella*, other antimicrobial factors occur (Table 6). One is a 60 kDa glycoprotein, termed aplysianin P [184,185]. This is bacteriostatic against various Gram-positive and Gram-negative bacteria at 0.2–5.8 μg mL^−1^ and seems to function by completely inhibiting the syntheses of bacterial DNA and RNA; a property that also lends it cytotoxic activity against eukaryotic tumour cells [184]. Related aplysianins are present in the albumin gland (aplysianin A) and eggs (aplysianin E) of *A. kurodai* [221,222]. The latter must certainly help the eggs from being colonized by bacterial and fungal epibionts. All of these aplysianins possess l-amino acid oxidase (LAAO) activity placing them within the family of LAAO enzymes. As the name suggests these deaminate l-amino acids in an oxidative manner to yield various microbicidal molecules, including H_2_O_2_, NH^+^ ions, carboxyl acids and α-keto acids [186,187,223]. A wide variety of LAAOs occur in marine and other organisms and most have broad-spectrum anti-infective properties [186]. One, designated *Sebastes schlegeli* antibacterial protein, is present in skin secretions of the rockfish, *Sebastes schlegeli*, and is active against the Gram-negative fish pathogens *A. hydrophila*, *A. salmonicida* and *Photobacterium damselae* ssp. *piscicida* [187] (Table 6). It has a yellow colouration and is derived from a 120 kDa homodimeric glycoprotein [187] (Table 6). Different LAAOs have different substrate specificity but the generation of the bactericidal products is usually very rapid [224]. One LAAO homolog of the aplysianins in sea hare ink is escapin, a 60 kDa monomer that has high oxidase activity with either l-lysine or l-arginine, and seems to kill actively growing bacteria by a variety of mechanisms, including hydrogen peroxide release [224]. Escapin is a highly stable compound that preferentially kills Gram-negative bacteria at MIC of ~0.25–0.62 μM mL^−1^, although it also prevents the growth of some Gram-positives, including *S. aureus* and has mild activity against certain fungi [224].

With micro-algae, several photosynthetic pigments or derivatives have been isolated and found to have microbicidal effects (Table 6). Jorgensen [188] reported the isolation of an antibacterial derivative of chlorophyll from extracts prepared from three different micro-algae (*Chlamydomonas reinhardi*, *Chlorella vulgaris*, *Scendesmus quadricauda*), which is active against the Gram-positive bacterium, *Bacillus subtilis*. The same compound, thought to be a photo-oxidation product of the chlorophyll derivative chlorophyllide *a*, was later isolated by Hansen [189] from acetone extracts of the chrysophyte, *Ochromonas malhamensis*. This antibacterial agent is active against Gram-positive and Gram-negative bacteria. In the same study, two further pigment derivatives with antibacterial activity were isolated but these could not be identified conclusively [189]. Finally, Bruce *et al*. [190] also reported the isolation of two chlorophyll *a* derivatives, which are active against Gram-positive and Gram-negative marine bacteria.

### 3.4. Pore-forming toxins

Many toxins have been identified from marine animals. Some groups of toxins share secondary structures with conventional AMP groups, whereas a small number have also been recorded to have antimicrobial activities. In addition, it is common for AMPs to gain access to the interior of a cell via pore-formation in the cytoplasmic membrane, which is a strategy employed by certain toxins. The best-known examples of these are the pardaxins of fish (Section 2.1.2) that were originally purified on the basis of their toxic, anti-predatory activity [48] yet have similar antibacterial potencies to the amphibian magainins and insect cecropins [50]

Secondary structures widespread in AMPS are also found in actinoporins, which are pore-forming toxins from the sea anemone, *Actinia equina* [191]. In particular, similarities exist between the actinoporin, equinotoxin II, and a major component of bee venom, melittin, that has strong antibacterial activity [225]. Similar to conventional AMPs, actinoporins contain an amphipathic region and a hydrophobic β-sandwich core (*i.e.*, a twisted β-sheet structure) with two adjacent α-helices [225]. The actinoporins appear to attach to membranes via the β-sandwich core while their actual pore-forming abilities appear to be mediated through the insertion of α-helices into membranes to form ion channels [226]. A recent review of membrane damage by proteins and toxins, including actinoporins, is given in Anderluh and Lakey [226]. Secondary structure similarities also exist between the tachystatins of horseshoe crabs and ω-agatoxins [66] of spiders (as discussed in Section 2.2.1). Other examples of pore-forming, α-helical toxins include the sticholysins from the sea anemone, *Stichodactyla helianthus* [192] and grammistins from the soapfishes, *Pogonoperca punctata* and *Grammistes sexlineatus* [193,194] (Table 6). The grammistins are active against Gram-positive (MIC 3.13 μg mL^−1^) and Gram-negative (MIC 6.25 μg mL^−1^) bacteria [194]. Although common structures are shared between AMPs and pore-forming toxins there are few reports concerning antimicrobial activities of the latter and this is an area worthy of further investigation.

### 3.5. Neuropeptides

Neuropeptides share several properties with AMPs, including amphipathicity, cationic charge and size. In addition, several neuropeptides (enkelytin, peptide B) and peptide hormones (e.g., neurotensin, bradykinin) from mammals have antimicrobial properties [227]. The antimicrobial activities of neuropeptides have not been widely investigated in fish or invertebrates [228], however there is evidence that neuropeptide fragments from the mussel, *M. edulis*, and the leech, *Theromyzon tessulatum*, possess antibacterial activities [195]. The opioid precursor pro-enkephalin A (PEA) releases smaller peptides when either animal species is challenged by LPS or physically stressed, e.g., via small surgical cuts [195]. The purified fragment, peptide B, from *M. edulis* is antimicrobial against Gram-positive bacteria [195] (Table 6).

### 3.6. Regulatory binding and other molecules

In addition to the unconventional anti-infectives derived from intracellular structures and pigments are a number of molecules that do not seem to be dedicated or professional AMPs, as they are often better known for functions other than defence. One is high-density lipoprotein (HDL), a component of mammalian blood serum. HDL has several physiological functions, but it is mainly associated with the transport of cholesterol and other lipids from atheroma of arteries to the liver. Along with its constituent apolipoproteins (apoAs) it further helps to inhibit damaging oxidative processes, regulate inflammation and modulate blood coagulation by affecting platelet aggregation. However, HDL and its apoA molecules also have antimicrobial properties [229–231] and appear to be cytotoxic to trypanosome parasites [232] (Table 6). The bactericidal activity of these molecules is likely to be due to the presence of amphipathic α-helices that enables them to attack the prokaryotic inner leaflet in a similar way to conventional α-helical AMPs. In mammals, serum HDL levels are only around 0.4–0.6 mg mL^−1^ and its contribution to non-specific immunity in mammals may be as a platform for the assembly of multi-component immune complexes [232]. By contrast, teleost fish have much higher levels of HDL, somewhere in the region of ~9–35 mg mL^−1^ [233] so HDL together with its derived apoAs could make a significant contribution to defence, especially as fish have greater reliance on innate immunity than warm blooded vertebrates. Indeed, HDL and apoAs from carp, trout and sea bass have been shown to have antibacterial activity against a range of bacteria [196,197,234,235], with inhibitory concentrations in the micromolar range [197]. HDL and its associated apoAs are also detectable and active in the skin epithelia of fish [198,234] revealing that they function as microbicidal effectors not only systemically but also in the mucosa.

### 3.7. Lectins

Invertebrates generally express different types of defence proteins in the blood plasma, of which many are involved in non-self recognition and in host defence. There are two types of molecule within the plasma of several protostome invertebrates that are primarily associated with recognition events, but that also appear to have antimicrobial effects (Table 6). One group, the lectins, bind sugars and are present in a wide variety of species, while the other group, anti-lipopolysaccharide factors (ALFs), are mainly found in arthropods, especially decapod crustaceans and horseshoe crabs, and bind LPS from Gram-negative bacteria.

Lectins are glycoproteins that bind specific carbohydrate moieties. A large number of lectins have been reported from marine invertebrates, often from immune cells or haemolymph [236]. Agglutination of bacteria is normally tested as part of lectin characterization, as agglutination may aid bacterial clearance by facilitating phagocytosis. However, parallel tests for direct bactericidal activity are frequently omitted from these analyses. A few reports exist of lectins with antimicrobial activity from marine macro-algae and invertebrates. Lectins from two red algal species, *Eucheuma serra* and *Galaxaura marginata*, inhibit the growth of *Vibrio vulnificus* and *V. pelagicus* although the inhibition was not recorded for all the *Vibrio* species tested and no Gram-positive bacteria were included in the screening [199] (Table 6).

Of marine invertebrate groups, antibacterial activity has been recorded for lectins from the sponges *Suberites domuncula* [200] and *Cliona varians* [201], the bivalve *Modiolus modiolus* [202], the crab, *Scylla serrata* [203], the horseshoe crab *T. tridentatus* [20] and the echinoderm *Holothuria scabra* [205] (Table 6). The *S. domuncula* lectin (designated LEC_SUBDO) was cloned from cultured *S. domuncula* cells using primers designed from two conserved regions of a galactose-binding protein from *T. tridentatus* and the native lectin was also purified from sponge cells [200]. Whereas native and recombinant LEC_SUBDO inhibit growth of *E. coli* by 81% and 36%, respectively, at a concentration of 300 μg mL^−1^, inhibition against the Gram-positive species *S. aureus* is much less (<15% inhibition) [200]. CvL, a lectin purified from the sponge, *C. varians*, is active against Gram-positive bacteria, inhibiting growth by 90% at 100 μg mL^−1^ [201]. Regarding bivalves, a sialic-acid binding lectin has been purified from the horse-mussel, *Modiolus modiolus*, which is particularly active against *Vibrio* species [202]. From crustaceans, a 5 kDa bactericidal lectin called scyllin has been purified from *S. serrata* [203]. Amongst the large group of horseshoe crab lectins, tachylectin 1 (originally called L6) from *T. tridentatus*, agglutinates both Gram-positive and Gram-negative bacteria but only inhibits the growth of Gram-negative species [204]. A recent study has purified a lectin (HSL) from the holothurian echinoderm, *Holothuria scabra*, which has activities against Gram-positive and Gram-negative bacteria [205] (Table 6).

The antimicrobial activities of fish lectins are less well established. Whilst some appear to inhibit bacteria this is not necessarily due to direct killing. An example is galectin from the eel, *Anguilla japonica*, that inhibits biofilm formation by human periodontopathic bacteria yet is not bactericidal [237]. A fish lectin that does have antibacterial activity, but only against *E. coli* when tested against a range of Gram-positive and Gram-negative bacteria, has been isolated from the ovary of the cobia, *Rachycentron canadum* [238].

### 3.8. Binding molecules

Anti-lipopolysaccharide factors (ALFs) are LPS-binding proteins that were discovered through their ability to inhibit LPS-induced clotting in the horseshoe crabs, *Tachypleus tridentatus* and *Limulus polyphemus* [239]. They are small basic peptides of ~100 amino acids that have strong affinity for negatively-charged surfaces, a feature that contributes to their LPS-binding and anti-coagulation properties [206]. High-resolution crystal analyses reveal that these molecules have a single domain of three α-helices packed against a four-stranded β-sheet, giving them a wedge-shaped appearance [240]. The molecules have a charge distribution that gives them striking amphipathicity and they interact with LPS by virtue of an amphipathic disulphide loop at its N-terminus [240–242].

However, in addition to their LPS-binding properties, ALFs also have strong microbicidal activities, mainly against Gram-negative bacteria [206] (Table 6). They are contained within the large granules of amoebocytes [64], unlike the more conventional antimicrobial peptides, e.g., tachyplesins, which are expressed in the small granules [243]. So they not only constitute a separate category of microbicidal proteins but are also segregated from them within the circulating blood cells. ALF molecules or genes have also been identified in shrimps [244–246], lobsters [247] and decapod crabs [19,248]. In all these species they are expressed and stored in the granules of circulating haemocytes and are rapidly released from the granules after exposure to bacterial LPS.

ALFs are one of a number of factors within the horseshoe crab clotting system that are extensively studied for their biopharmaceutical value. So far interest has lain mainly in their value as endotoxin-neutralising agents to prevent or mitigate septic shock in patients with serious bacteraemia but they also appear to have other pharmacological effects on mammalian immune cells, including modulation of cytokine expression [249,250]. Examples of the effects of this molecule include inhibition of TNFα production from mammalian leucocytes, inhibition of nitrate formation by LPS-activated murine macrophages and activation of human mononuclear cells to release antiviral proteins (mainly interferons) [249,250]. These properties, combined with their microbicidal effects on certain human pathogens, illustrate well the potential value of novel compounds from marine invertebrates.

## 4. Discussion

As is evident from this brief survey of anti-infective agents from marine or aquatic fish, invertebrates and micro-algae, these eukaryotic organisms express a very wide range of compounds including not only conventional, (*i.e.*, ‘dedicated’) antimicrobial peptides within the immune system but also a large array of proteins, protein fragments, lipoproteins, glycoproteins or other factors that have, or are derived from, compounds with other primary biological functions. The sheer number of different types expressed even within a single species is quite remarkable, and could not have been imagined thirty or forty years ago, before the discovery of AMPs stimulated prospecting for novel antibiotics from natural sources. Why do so many molecules have, or appear to have, antibiotic effects? Do they all have, or have had at some point in evolution, some survival benefits against micro-organisms for the host? Are they also of potential value to us as novel anti-infective drugs or treatments? Importantly, too, are there other compounds, not currently considered as direct innate immune effectors, made by marine or aquatic organisms that possess antimicrobial properties and could show promise for future medical or commercial exploitation?

### 4.1. Diversity and evolution of antibacterial molecules

It is possible only to speculate as to why so many different molecules in eukaryotic organisms have the ability to kill micro-organisms. Obviously the threat to eukaryotes from prokaryotic competitors is very great, especially in the sea where microbes abound and nutrients are generally low. Even the earliest eukaryotes in evolution would have needed to protect themselves from prokaryotic domination. Small peptides are generally considered to be the most ancient of microbicidal agents because they are so ubiquitous and their simple molecular configuration makes them inexpensive to produce, even by organisms without specialized tissues and defence cells. It is plausible that certain functional molecular motifs, such as amphipathic α-helices and β-sheets, would have featured predominantly in the arsenal employed by our distant eukaryotic ancestors. Over time, these would have diversified through mutation and selection pressure from bacterial resistance to produce the great variety of AMPs and other molecules that exist today across so many different molecules in widely divergent taxa.

Certainly, it is curious that many of the unconventional antimicrobial agents described above possess amphipathic structures, typically α-helices or β-sheets, even though they are expressed at sites that would not necessarily bring them into close contact with invading micro-organisms. A breakthrough discovery in 2004 by Brinkmann *et al*. [251] changed this by revealing a new cell death pathway, distinct from necrosis or apoptosis, which operates in inflammation and provides a mechanism by which histones and other intracellular proteins may be presented to bacteria or other infective agents. This is a process by which chromatin de-condenses but does not fragment within the nucleus, and as the nuclear membrane breaks down, is expelled explosively from the cell to form an extracellular net or mesh [251–253]. These nets entrap bacteria preventing their spread and facilitate their killing by histones and cationic AMPs that characteristically stud the chromatin meshes [251–253]. This new cell death process, now termed ETosis (because it is due to the formation of extracellular traps), was observed initially in mammalian neutrophils but has subsequently been reported for mammalian mast cells [254] and eosinophils [255] (see also [253]). Circumstantial evidence has also been presented that neutrophils from zebrafish and fathead minnow exhibit a similar process [256,257], as do phagocytes from insects [258] and marine crabs (Roulston, Robb, Rossi, Dyrynda and Smith, unpublished observations), although further work is needed to confirm that these invertebrate nets are equivalent to those produced by mammalian neutrophils. It would be interesting to establish if ETosis is also performed by the wandering, mesogleal phagocytes of acoelomates or even single-celled eukaryotes, such as micro-algae. If ETosis is indeed a very ancient process, it would enable us to regard histones, ribosomal peptides, membrane-derived free fatty acids or other antimicrobials derived from intracellular structures not as ‘unusual’ or ‘unconventional’ effectors but as primordial ones for the Eukaryota as a whole. With this information, we would then have a much clearer idea whether or not, at some point in evolution, apparently unusual anti-infective molecules had some survival benefits against micro-organisms for the host.

### 4.2. Potential value of marine eukaryotic anti-infectives

With respect to the question regarding the potential value of the anti-infective agents already described for marine and aquatic organisms in clinical, veterinary or biotechnological application, the jury is still out. So far, relatively few have received commercial consideration; the clotting and ALFs from horseshoe crabs being the notable exceptions (although it should be noted that these have been known and studied for over 20 years). Clearly many new antimicrobial agents are being discovered in marine animals and the growing list of such antimicrobial proteins now on databases or described in learned journals reflects the huge input of research time and money that are made available for bio-prospecting projects. The more we look, the more we find, although at this stage it is hard to know which of these novel antimicrobials might endure and become market successes. Many may not pass muster because of problems of toxicity, antigenicity and production costs. Others might show initial promise but have poor stability within the mammalian body, be unpalatable and therefore of limited use in food preservation or they may be ineffective against the most problematic pathogens that affect human society. Another problem is whether patent rights would be possible for some ubiquitous well-known compounds, such as free fatty acids and histones, even though they may score highly on potency, stability and patient toleration. Notwithstanding these issues, the use of histone H1 as a synthetic additive to pharamaceutical or nutraceutical formulations or kits has already been patented [259].

Certainly, the available information for many marine anti-infectives from eukaryotes is patchy. Potency measures vary from author to author as do the choice of test micro-organisms and the tissue or cells from which extracts are made. Thus it is difficult to make comparisons between compounds at least in relation to efficacy. In terms of drug development, what also matters are the assay conditions under which antimicrobial activity is measured. The tissues of marine eukaryotes, especially invertebrates and micro-algae, are often iso-osmotic to seawater, so any antibacterial compounds they synthesise might be expected to function under the high salt and slightly alkaline conditions of their environment if they are to serve a useful purpose in defence for their host. However, it is well known that many antimicrobials lose their activity under high salt or inappropriate pH conditions, usually as a consequence of alterations in charge, folding and amphipathicity of the active molecule [44,260,261]. Indeed the effect of salt is a particular issue in the development of new antibiotics for topical application (*i.e.*, on the skin) as sweat creates a hyper-osmotic environment. It is therefore important in the discovery of new anti-infectives that microbicidal activity is assessed under conditions both physiologically relevant to the host from which it was sourced as well as those under which it might be deployed in commercial or clinical use. Salt stability is a desirable characteristic to obtain or engineer into new drugs although relatively few natural anti-infectives are known to have a high salt requirement. One compound that does require high salt concentrations is the 11.5 kDa crustin, carcinin, from the shore crab, *Carcinus maenas* [82]. This protein loses its activity if it is assayed in buffers containing 0.7 M NaCl even against a marine bacterium that is normally very susceptible to its effects but has been acclimated to low salt conditions by repeat sub-culturing into media of reduced salinities [82]. At present the reason for this salt requirement is unknown but for some other anti-infectives progress has been achieved in modifying the active molecules, for instance by helix capping motifs [262] or by inclusion of cyclic tricystine structures [261], to improve their performance in high saline conditions. Additional, valuable information about other strategies that could address problems such as salt stability might emerge from further analyses of natural peptide antibiotics from marine animals.

### 4.3. Promise for future medical or commercial exploitation

For the future, prospecting projects on marine fish, invertebrates and micro-algae might offer insights not only into how salt stability of antibiotics may be improved but also in expanding our knowledge of the diversity of chemical structures that bring about bacterial killing. The way amphipathic structures kill bacteria are well understood [35,36] but alternate processes that work, not just at the membrane but within the bacterial cell, are less so. For example, histone H2A-derived fragments seem to bind to and interact with bacterial DNA [263]. Such processes need deeper analysis and others may be discovered that have completely novel ways of inactivating or killing bacterial cells.

#### 4.3.1. Synergy

Importantly, too, little effort has been exerted into exploring the use of anti-infectives from marine eukaryotes in combination therapies with traditional antibiotics. Certainly synergy between different antimicrobials produced within the same organism must occur in nature, and there are a few sporadic reports of synergism occurring between co-expressed proteins in some fish or invertebrates. For example, with invertebrates, Gueguen *et al*. [97] used an *in vitro* checkerboard assay to study synergy between recombinant defensin and synthetic fragments of a 61-residue proline-rich AMP from the oyster, *C. gigas*. The combination of these agents produced around a threefold increase in the killing of *E. coli* [97]. Synergism between tachycitin and big defensin, two conventional antimicrobial peptides from amoebocytes of horseshoe crab, *Tachypleus tridentatus*, has also been reported [264]. As little as 0.9 μg mL^−1^ tachycitin reduces the IC_50_ of big defensin from 0.8 μg mL^−1^ to 0.015 μg mL^−1^ [264].

Lysozyme is another important anti-infective in a wide range of coelomate animals that can synergize with other anti-infectives. It occurs very widely across the animal kingdom, often in multiple forms and has the effect of breaking open the peptidoglycan cell wall of Gram-positive bacteria, thus allowing AMPs to enter the cell and permeabilize the inner leaflet. Accordingly it can serve both as a direct effector and as synergiser of AMP activity. Notably, teleost fish possess multiple types of lysozyme-like muramidases, especially in skin secretions. These include not only the conventional cationic lysozymes but also an unusual anionic type of muramidase, which has yet to be fully characterised [265]. Concha *et al*. [197] has also reported that lysozyme enhances the antimicrobial ability of apoA-1 from carp, and a further example of synergism between different antimicrobial proteins is given by the work of Patrzykat *et al*. [103]. These authors combined native pleurocidin with lysozyme, HSDF-1 or HSDF-2 (two different fragments of histone H1 from Coho salmon). These combinations produced highly synergistic improvements in inhibiting the growth of the fish pathogens, *Aeromonas salmonicida* and *L. anguillarum* [103] and shows that histones also have potential to be deployed as synergists for other antibiotic substances. Histones are known to disrupt lipid bilayers but do not seem to form pores in bacteria and do not have haemolytic effects [105,175] so could be well tolerated by patients receiving such treatments.

Oxylipins and FFAs, too, might find application in combination therapies, as synergists for non-lipid anti-infectives, including some of those described above. Unfortunately few studies have investigated the potential of oxylipins, despite their known activities against important pathogens [172] (Table 5). By contrast FFAs are already on the agenda for exploitation as bio-pharmaceuticals alone or in synergy with anti-infectives for use in medicine [159,266,267]. The great advantage of FFAs lies in their broad-spectra of activity, high potency, relative safety and the lack of inducible resistant phenotypes [134,268–271]. Those from micro-algal extracts should be of high quality, cheap and easy to produce on a commercial scale and have already shown particular promise in formulations to prevent the colonisation of human skin by opportunistic pathogens, such as MRSA [272]. Fatty acid-enriched gels have been produced that may be useful in preventing the spread of sexual transmitted bacterial and viral pathogens [273], while the palmitoleic acid isomer, C16:1n-10, has been found to be effective at reducing systemic staphylococcal burden in mice and therefore might prove valuable for the treatment of serious systemic infections of humans by *S. aureus* and MRSA [274].

#### 4.3.2. Chimeric compounds

**Figure 1:**
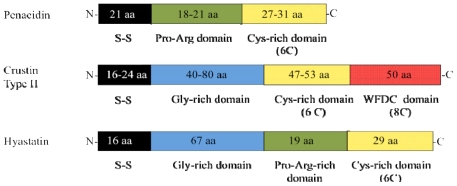
Schematic representation of chimeric domain organisation within penaeidins, crustin type II AMPs and hyastatin, three conventional AMPs from crustaceans. WFDC, whey four disulphide core containing domain; S-S, signal sequence. The number of cysteine residues (C) within the cysteine rich domains is indicated in parentheses. Data from Destoumieux *et al*. [276]; Smith *et al*. [14] and Sperstad *et al*. [16].

A final word might be said about the possibility of using knowledge obtained about the structures and gene organisation of marine-derived anti-infectives to design chimeric compounds that effectively combine synergistic or additive motifs into a single molecule or drug. Some recent work on conventional AMPs from crustaceans has revealed that nature has already created some multi-domain AMPs, each domain of which being known from other research to have independent, potent antimicrobial properties. Good examples are afforded by hyastatin from the spider crab, *Hyas arenaeus* [16] and penaeidins from shrimp [17]. The penaeidin domains comprise a proline-arginine-rich region at the N-terminus and a cysteine-rich one at the C-terminus [275] (Figure 1). The proline-rich region is unstructured and forms a long tail, in contrast to the highly ordered conformation of the C-terminus, which is tightly coiled with an α-helix and three disulphide bonds [277]. The chitin-binding activity of the C-terminus is likely to contribute to the antifungal properties of the penaeidins, and may also allow the peptide to become anchored to the carapace during wound healing or at the moult [276]. The N-terminus proline-arginine-rich domain, however, seems to account for the antibacterial properties of the whole molecule [278].

The second example from crustaceans, hyastatin, is made up of three domains and, like penaeidins and many conventional AMPs, is liberated from its inactive precursor by proteolytic cleavage of the N-terminus signal sequence [16] (Figure 1).

The N-terminus of the mature protein has a glycine-rich (LGGG/IGGG) domain and a C-terminus cysteine-rich domain with six cysteine residues that probably form an α-helical configuration with three disulphide bonds, while in between the termini is a short proline-arginine-rich region [16] (Figure 1). The glycine-rich domain has a striking resemblance to the glycine-rich domain of shrimp Type II crustins while the C-terminus and central proline-arginine domains are similar to the proline-arginine- and cysteine-rich domains of penaeidins [16]. Thus hyastatin appears to be chimeric, comprising domains that characterise and account for the functionality of other, quite distinct anti-infective proteins in separate crustacean groups. Interestingly, hyastatin seems to owe its antibacterial properties to the cysteine-rich C-terminus domain, like crustins [14], because a recombinant protein lacking the cysteine-rich domain has no bactericidal activity [16]. However, as mentioned earlier when considering conventional, amino acid enriched AMPs, the chitin-binding properties may indicate that the peptide is multifunctional. Nature thus seems to ‘mix and match’ useful domains to create AMPs that could have dual mechanisms to destroy their targets. The possibility remains that synthetic anti-infectives might be designed and engineered that are also chimeric but use structures known to have particular efficacy against certain types of pathogen to maximize killing and reduce the risk of resistance.

## Figures and Tables

**Table 1:** Major conventional AMPs/AMP families isolated from marine invertebrates and fish: distribution across phyla.

Taxon	Peptide/peptide family	Key reference

**Cnidaria (Scyphozoa)**	Aurelin	Ovchinnikova *et al.* [6]

**Annelida (Polychaeta)**	Arenicin	Ovchinnikova *et al.* [7]
	Hedistin	Tasiemski *et al.* [8]
	Perinerin	Pan *et al.* [9]

**Mollusca (Bivalvia)**	Big defensins 1	Li *et al.* [10]
	*Cg*-prp	Li *et al.* [10]
	Defensins 1	Li *et al.* [10]
	Myticins 1	Li *et al.* [10]
	Mytilins 1	Li *et al.* [10]
	Mytimycin	Li *et al.* [10]

**Crustacea (Decapoda)**	Arasin-1	Stensvåg *et al.* [11]
	Bac-like	Schnapp *et al.* [12]
	Callinectin	Khoo *et al.* [13]
	Crustins 1	Smith *et al.* [14]
	Homarin	Battison *et al.* [15]
	Hyastatin	Spersted *et al.* [16]
	Penaeidins 1	Cuthbertson *et al.* [17]
	Scygonadin	Huang *et al.* [18]
	*Scylla serrata* antimicrobial protein	Yedery and Reddy [19]

**Chelicerata**	Big defensin	Saito *et al.* [20]
	Polyphemusins 1	Miyata *et al.* [21]
	Tachycitin	Kawabata *et al.* [22]
	Tachyplesins 1	Miyata *et al.* [21]
	Tachystatins 1	Osaki *et al.* [23]

**Echinodermata (Echinoidea)**	Strongylocins 1	Li *et al.* [24]

**Urochordata (Ascidiacea)**	Clavanins 1	Lee *et al.* [25]
	Dicynthaurin	Lee *et al.* [26]
	Halocidin	Jang *et al.* [27]
	Halocyamines 1	Azumi *et al.* [28]
	Halycyntin	Galinier *et al*. [29]
	Papillosin	Galinier *et al.* [29]
	Styelins 1	Lee *et al*. [30]

**Chordata (Pisces)**	Cathelicidins 1	Smith and Fernandes [31]
	Defensins 1	Smith and Fernandes [31]
	Hepcidins	Smith and Fernandes [31]
	Liver-expressed antimicrobial peptides (LEAPs) 1	Smith and Fernandes [31]
	Piscidins 1	Smith and Fernandes [31]

1 Family of peptides as opposed to a single novel protein.

**Table 2:** Distribution of conventional AMPs/AMP families into structural categories.

Categories of AMPs		Examples	Activity 1
Linear, α-helical		Clavanins (Ascidians)	G+, G−, F
		Dicynthaurin (Ascidians)	G+, G−, H
		Halocyntin (Ascidians)	G+, G−
		Papillosin (Ascidians)	G+, G−
		Piscidins (Fish)	G+, G−, F, H
		Styelins (Ascidians)	G+, G−, H
Cysteine-rich	No. disulphide bonds		
2		Cathelicidins (Fish)	G+, G−
		LEAPs (Fish)	G−, F
		Tachyplesins (Horseshoe crabs)	G+, G−, F
		Polyphemusins (Horseshoe crabs)	G+, G−, F
3		Aurelin (Jellyfish)	G+, G−
		Big defensins (Horseshoe crabs)	G+, G−, F
		Penaeidins (Shrimp)	G+, G−, F, Cb
		Strongylocins (Sea urchins)	G+, G−
		Tachystatin (Horseshoe crabs)	G+, G−, F, H 2, Cb
4		Defensins (Molluscs)	G+, G−3, F
		LEAPs (Fish)	G−, F
		Myticins (Molluscs)	G+, G−4, F 4
		Mytilins (Molluscs)	G+, G−, F 5
		Crustins (WAP domain; crabs)	G+
5		Tachycitin (Horseshoe crabs)	G+, G−, F, Cb
Cationic peptides: specific amino acid enriched		Arasin-1 (Spider crab) (proline and arginine rich) Bac-like (Crab) (proline rich) *Cg*-prp (Oyster) (proline rich) Hyastatin (Spider crab) (glycine rich) Penaeidins (Shrimp) (proline and cysteine rich)	G+, G− G+, G− Synergises with defensin G+, G−, F, Cb G+, F, Cb
Miscellaneous		Arenicin (Polychaete) Hedistin (Polychaete) Perinerin (Polychaete)	G+, G−, F G+, G− G+, G−, F

1 G+, Gram-positive; G−, Gram-negative; F, Fungi; H, haemolytic; Cb, chitin-binding;

2 Tachystatin C only;

3 Defensin from *Crassostrea virginica*;

4 Myticin B isoform;

5 Mytilin isoforms B & D.

**Table 3:** Unconventional antimicrobial proteins and peptides derived from intracellular structures of fish and invertebrates.

Protein/peptide	Location	Active factor	Reported activity 1	Source	Key reference

Histone H1	Nucleus	Whole protein (20.7 kDa)	G−	Salmon	Richards *et al.* [104]
		N-terminus (26 aa) (HSDF-1)	G−	Salmon	Patrzykat *et al*. [103]
		C-terminus (69 aa) (oncorhyncin II)	G+, G−	Rainbow trout	Fernandes *et al*. [107]
		Fragment (not specified)	G+	Shrimp	Patat *et al*. [108]

Histone H2A	Nucleus	Whole protein (13.5 kDa)	G+, F	Channel catfish	Robinette *et al*. [102]
		Whole protein (13.5 kDa)	G+, H	Rainbow trout	Fernandes *et al*. [105]
		Whole protein (13.5 kDa)	G+, G−	Shrimp	Patat *et al*. [108]
		N-terminus (51 aa) (hipposin)	G+, G−	Halibut	Birkemo *et al*. [109]
		N-terminus (19 aa) (parasin-1)	G+, G−, F	Catfish	Park *et al*. [101]
		N-terminus (40 aa) (abhisin)	G+, F, Cy	Abalone	De Zoysa *et al*. [110]

Histone H2B	Nucleus	Whole protein (13.8 kDa)	G−	Cod	Bergsson *et al*. [111]
		Whole protein (15.5 kDa)	G−, F	Channel catfish	Robinette *et al.* [102]
		Whole protein (13.5 kDa)	G+	Shrimp	Patat *et al*. [108]

Histone H3	Nucleus	Whole protein (15.3 kDa)	G+	Shrimp	Patat *et al*. [108]

Histone H4	Nucleus	Whole protein (11.3 kDa)	G+	Shrimp	Patat *et al*. [108]

HMG H6	Nucleus	Whole protein (6.7 kDa) (oncorhyncin III)	G+, G−	Fish	Fernandes *et al*. [112]

40Rsp30	Ribosomes	Whole protein (6.7 kDa)	G+	Rainbow trout	Fernandes and Smith [113]
60RspL40		Whole protein (6.4 kDa)	G+, G−	Cod	Bergsson *et al*. [111]
60RspL36A		Whole protein (12.3 kDa)	G+ G−	Cod	Bergsson *et al*. [111]
60RspL35		Whole protein (14.2 kDa)	G+	Cod	Bergsson *et al*. [111]

1 G+, Gram-positive; G−, Gram-negative; F, Fungi; H, haemolytic; Cy, cytotoxic.

**Table 4:** Effect of structure on the antibacterial activity of free fatty acids. Antibacterial activity was assessed by disc diffusion against the Gram-positive bacterium *Bacillus larvae* with 250 μg of compound per sterile paper disc. The area of microbial growth inhibition (clear zone area) was calculated as total area of clear zone minus the area of the disc. Larger clear zones indicate greater antibacterial activity. Data modified from Feldlaufer *et al*. [132].

Structural feature	Fatty acid	Clear zone area (mm^2^)

**Carbon chain length: saturated fatty acids**	C6:0	0
	C8:0	223
	C9:0	1230
	C10:0	2260
	C11:0	2800
	C12:0	5000
	C13:0	1230
	C14:0	46.9
	C15:0	0
	C16:0	0
	C17:0	0
	C18:0	0

**Carbon chain length: monounsaturated fatty acids**	C14:1 n-5	5000
	C16:1 n-7	4040
	C18:1 n-9	0
	C20:1 n-9*t*	584
	C22:1 n-9	0

**Degree of unsaturation**	C22:1 n-9	0
	C22:2 n-6	584
	C22:3 n-3	1230
	C22:4 n-6	1930
	C22:6 n-3	2090

**Bond orientation(s)**	C16:1 n-7	4040
	C16:1 n-7*t*	675
	C18:2 n-9	3600
	C18:2 n-9*t*^1^	1230

1 Both bonds in *trans* orientation.

**Table 5:** Susceptibility of marine bacteria and potential human or animal pathogens to the antibacterial effect of eicosapentaenoic acid (EPA) or decadienal (DD). Antibacterial activity was assessed by disc diffusion [149] with 1 μM of compound per sterile paper disc. The area of microbial growth inhibition was calculated as defined in the legend for Table 4.

Species, strain and Gram’s stain 1	Clear zone area (mm^2^)	
	DD	EPA

**Marine isolates**		
*Aeromonas hydrophila* NCIMB 1108(G−)	483	0.0
*Alteromonas haloplanktis* NCIMB 19 (G−)	22.0	0.0
*Listonella anguillarum* MT 1637 (G−)	566	15.9
*Photobacteriumphosphoreum* NCIMB 64 (G−)	22.0	22.0
*Psychrobacter immobilisNCIMB 308* (G−)	84.8	0.0
*Micrococcus luteus* NCIMB 9278 (G+)	42.6	50.3
*Planococcus citreus* NCIMB 1493 (G+)	1600	50.3

**Opportunistic human and animal pathogens**		
*Escherichia coli* B (G−)	18.9	0.0
*Pseudomonas aeroginosa* NCIMB 10775 (G−)	10.2	0.0
*Staphylococcus aureus* SH 1000 (G+)	50.3	173
*Staphylococcus epidermidis* ATCC 10145 (G+)	22.0	105

1 G+, Gram-positive; G−, Gram-negative.

**Table 6:** Examples of other anti-infective compounds or fragments derived from these compounds, which serve alternative functions in marine organisms.

Name	Main function	Size	Activities 1	Organism(s)	Key references

**Respiratory pigments**					
Haemoglobin fragments	Respiratory pigment	28, 41 kDa	G+, G−	Rainbow trout	Fernandes and Smith [174]
Haemocyanin fragments	Respiratory pigment	7.9, 8.3 kDa	F	Shrimp	Destoumieux *et al.* [175]

**Other pigments**					
Echinochrome A	Blood pigment	266 Da	G+, G−	Sea urchins	Service and Wardlaw [176]
Melanin	Blood pigment	~318 kDa	G+, G−, F	Crustaceans	Söderhäll and Ajaxon [177]; Nappi and Ottaviani [178]; Lin and Chen [179]; Burkhart and Burkhart [180]
Melanin	Ink pigment	~318 kDa	G+, G−, F	Octopus	Prota *et al.* [181]
Prophenoloxidase	Enzyme	60–77 kDa	F	Solitary ascidian	Hata *et al.* [182]
Prophenoloxidase	Enzyme	60–77 kDa	G+, G−	*Amphioxus*	Li *et al.* [183]
Aplysianins	Ink component	60–320 kDa	G+, G−, F, Cy	Sea hares	Yamazaki *et al.* [184]
Dolabellin	Ink component	60 kDa	G+, G−, Cy	Sea hares	Yamazaki *et al.* [185]
l-amino acid oxidases	Ink component	340 kDa	G+, G−	Many organisms	Derby [186]
*Sebastes schlegeli* antibacterial protein (l-amino acid oxidase)	Skin mucus	120 kDa	G−	Fish	Kitani et al. [187]
Chlorophyll derivatives	Photosynthetic pigment	~0.6 kDa	G+, G−	Various micro-algae	Jorgensen [188]; Hansen [189]; Bruce *et al*. [190]

**Pore-forming toxins**					
Pardaxin	Skin toxin	3–4 kDa	G+, G−, H	Flatfish	Lazarovici *et al*. [48]
Actinoporins	Skin toxin	20 kDa	H, Cy	Sea anemones	Kristan *et al.* [191]
Sticholysins	Skin toxin	20 kDa	H, Cy	Sea anemones	Alvarez et al. [192]
Grammistins	Skin toxin	~1–3 kDa	G+, G−	Soapfish	Yokota *et al.* [193]; Sugiyama *et al.* [194]

**Neuropeptides**					
Peptide B	Neuropeptide fragment	2.5–3.5 kDa	G+	Mussel	Tasiemski *et al*. [195]

**Regulatory factors**					
HDL/ApoA-1	Various functions	29.5 kDa	G+, G−	Carp	Concha *et al.* [196]
HDL/ApoA-1	Various functions	29.5 kDa	G+, G−	Rainbow trout	Villaroel *et al.* [197]

**Lectins**					
ESA	Lectin	Not specified	G−	Red alga	Liao *et al.* [198]
GMA	Lectin	Not specified	G−	Red alga	Liao *et al.* [198]
LEC_SUBDO	Lectin	27 kDa	G+, G−	Sponge	Schroeder *et al*. [199]
CvL	Lectin	106 kDa	G+	Sponge	Moura *et al*. [200]
Sialic-acid binding lectin	Lectin	~51 kDa	G−	Bivalve mollusc	Tunkijjanukij and Olafsen [201]
Scyllin	Lectin	5 kDa	G+, G−	Mud crab	Chattopadhyay and Chatterjee [202]
Tachylectin-1	Lectin	27 kDa	G−	Horseshoe crabs	Saito *et al*. [203]
HSL	Lectin	182 kDa	G+, G−	Holothurian	Gowda *et al*. [204]

**Binding molecules**					
Anti-lipopolysaccharide factor (ALF)	Endotoxin-binding protein	~15 kDa	G−	Horseshoe crabs	Morita *et al.* [205]

1 G+, Gram-positive; G−, Gram-negative; F, Fungi; H, haemolytic; Cy, cytotoxic.
